# ﻿*Hemiaustroboletus*, a new genus in the subfamily Austroboletoideae (Boletaceae, Boletales)

**DOI:** 10.3897/mycokeys.88.73951

**Published:** 2022-03-30

**Authors:** Olivia Ayala-Vásquez, Jesús García-Jiménez, Elvira Aguirre-Acosta, Rigoberto Castro-Rivera, Rodolfo Enrique Ángeles-Argáiz, Ángel Emmanuel Saldivar, Roberto Garibay-Orijel

**Affiliations:** 1 Tecnológico Nacional de México, Instituto Tecnológico de Ciudad Victoria, Blvd. Emilio Portes Gil #1301 Pte., Ciudad Victoria, Tamaulipas, CP 87010, Mexico Tecnológico Nacional de México, Instituto Tecnológico de Ciudad Victoria Ciudad Victoria Mexico; 2 Instituto de Biología, Universidad Nacional Autónoma de México, Circuito exterior s/n Ciudad Universitaria, Ciudad de México, CP 04510, Mexico Universidad Nacional Autónoma de México Ciudad de México Mexico; 3 CIBA, Instituto Politécnico Nacional, Tlaxcala, CP 90700, Mexico CIBA, Instituto Politécnico Nacional Tlaxcala Mexico; 4 Departamento de Botánica y Zoología, Universidad de Guadalajara, Zapopan, Jalisco, CP 45101, Mexico Universidad de Guadalajara Jalisco Mexico

**Keywords:** Mexico, mycodiversity, neotropics, new taxa

## Abstract

The present study describes *Hemiaustroboletus***gen. nov.** in the subfamily Austroboletoideae (Boletaceae). *Hemiaustroboletus* is supported by morphological and molecular data using LSU and RPB2 regions. Additionally, its geographic distribution and intraspecific variation were inferred using ITS sequences. The genus is characterised by pileate-stipitate basidiomata; purple, brown, reddish-brown, orange-brown to dark brown vinaceous pileus; whitish or lilac to vinaceous context and a subclavate stipe. Microscopically, it is characterised by ornamented, slightly verrucose, cracked to perforated brown basidiospores. Two species are described within the genus, *Hemiaustroboletusvinaceobrunneus***sp. nov.** and *H.vinaceus***sp. nov.***Hemiaustroboletusvinaceus***sp. nov.** is morphologically similar to *Austroboletusgracilis*, which suggests they may have been confused in the past. This study presents the phylogenetic placement, microscopic structures, detailed morphological descriptions and illustrations of both new species.

## ﻿Introduction

Boletaceae is the most diverse family within the Boletales; it has a wide distribution in both temperate and tropical regions (Binder and Hibbett 2006; Wu et al. 2014). Most species of this family are ectomycorrhizal with members of Betulaceae, Casuarinaceae, Dipterocarpaceae, Ericaceae, Fabaceae, Fagaceae, Mimosaceae, Myrtaceae, Pinaceae, Polygonaceae, and Salicaceae (Tedersoo et al. 2010; Smith et al. 2013; Wu et al. 2016). Currently, 98 genera are recognised in this family (He et al. 2019; Vadthanarat et al. 2019; Hosen and Yang 2021). Its members are characterised by fleshy, epigeous pileate-stipitate basidiomata or hypogeous to subhypogeous gastroid basidiomata, with tubular or lamellar hymenophore; elliptical, cylindrical, fusoid, subfusoid, ovoid, subglobose to globose, smooth or ornamented basidiospores; spore ornamentation ranging from striated, reticulate, echinulate, filiform and perforated to verrucose (Singer et al. 1991; Wu et al. 2014; Halling et al. 2015; Ayala-Vásquez et al. 2018).

Wu et al. (2014) proposed six subfamilies for Boletaceae, of which Austroboletoideae includes *Austroboletus* (Corner) Wolfe, *Fistulinella* Henn., *Mucilopilus* Wolfe and *Veloporphyrellus* L.D. Gómez & Singer, with *Austroboletus* as the type genus. This subfamily is distinguished by pileate-stipitate basidiomes; smooth, furfuraceous, tomentose, dry or viscous pileus, with or without a marginal veil and whitish context that does not change colour when cut. The hymenophore is tubular, whitish or pink with purple tinge, immutable or rarely brown when cut. The stipe is smooth, reticulate or squamose with a whitish basal mycelium. The basidiospores are smooth or ornamented, perforated, verrucose to smooth, grey-violet, yellowish, yellow brown, ochraceous in potassium hydroxide (KOH) and yellow-brown, yellow-cinnamon to ochraceous in Melzer’s reagent. The pileipellis is formed by a trichoderm or ixotrichoderm. The hymenophoral trama is boletoid. Austroboletoideae species are mainly associated with Fagaceae and Pinaceae hosts in temperate, subtropical to tropical regions.

In recent years, various authors (Wu et al. 2014; Wu et al. 2016; Gelardi et al. 2020; Kuo and Ortiz-Santana 2020) have recognised the polyphyly of *Austroboletus*, which is divided into the *Austroboletus* s.s., *Austroboletus* s.l. and the *A.gracilis* s.l. independent clades. This study focuses on the phylogenetic placement and taxonomy of the *A.gracilis* s.l. clade, placing it in the new genus *Hemiaustroboletus* with two new species, *Hemiaustroboletusvinaceobrunneus* and *H.vinaceus*.

## ﻿Materials and methods

To resolve the systematics and taxonomy of the new genus *Hemiaustroboletus*, we conducted an exhaustive sampling of an area with high bolete diversity according to García-Jiménez et al. (2013). The sampling was carried out over the last 10 years including the different biogeographic areas of Mexico: Nearctic, Neovolcanic Axis and Neotropic. The collection trips were conducted in the States of Chiapas, Chihuahua, Estado de Mexico, Jalisco, Michoacan and Oaxaca, in six vegetation types in temperate and subtropical forests during the rainy season from June to October from 2010 to 2019. The samples were characterised at macro- and micromorphological level and three genetic markers were sequenced and analysed.

### ﻿Morphological study

Morphological characters were described according to Largent (1986) and Lodge et al. (2004). Chemical reactions with KOH and ammonium hydroxide (NH₄OH) were characterised. Photographs of basidiomata were taken *in situ*, as well as data on the botanical composition of the sites. The colours for taxonomic descriptions were based on Kornerup and Wanscher (1978). Microscopic characters of 30 basidiospores, basidia, pleurocystidia, cheilocystidia, pileipellis cells and stipitipellis were measured by optical microscopy (Carl Zeiss GmbH 37081, Germany). The Q index (length/width) was estimated for the basidiospores. Ornamentation of basidiospores was observed by scanning electron microscopy (SEM) (Hitachi Su 1510, Hitachi, Japan). The specimens were deposited at the “Herbario Nacional de México” of the “Instituto de Biología, Universidad Nacional Autónoma de México” (MEXU), at the “Herbario José Castillo Tovar del Tecnológico de Ciudad Victoria” (ITCV) and at the “Herbario del Instituto de Botánica, Universidad de Guadalajara” (IBUG).

### ﻿DNA Extraction, PCR and Sequencing

Samples of dehydrated basidiomata were used for DNA extraction. The DNA was extracted using the DNeasy Power-Soil kit (QIAGEN). Cell lysis was performed by grinding samples in mortar with liquid nitrogen. Three nuclear loci (ITS, LSU and RPB2) were amplified with Platinum Taq DNA Polymerase (Invitrogen-Thermo Fisher Scientific) and Taq & Load PCR Mastermix (MP Biomedicals) in a thermocycler (BIO-RAD). The PCR parameters were as follows: 95 °C initial denaturation for 4 min; 35 cycles of denaturation at 94 °C for 60 s, alignment at 54 °C for 60 s, extension at 72 °C for 60 s and a final extension at 72 °C for 10 min. The primers ITS1/ITS4 (White et al. 1990) were used for the ITS region; LROR/LR5 (Vilgalys and Hester 1990) for LSU; and RPB2-B-F2/RPB2-B-R (Wu et al. 2014) for the partial RPB2 gene. The amplification was examined by 1% agarose gel electrophoresis; gels were stained with GelRed (Biotium) and observed under an UVP Multidoc-It transilluminator (Analytikjena). Only PCR products generated with Taq-Platinum required LB loading buffer. PCR products with successful amplification were cleaned with ExoSAP-IT (Thermo Fisher Scientific) diluted 1:1 with ddH_2_O and incubated at 37 °C for 45 min and 80 °C for 15 min. Sanger sequencing was performed at the “Laboratorio de secuenciación genómica de la biodiversidad y la salud, Instituto de Biología, Universidad Nacional Autónoma de México”. Samples were sequenced in both directions with PCR primers using BigDye Terminator v.3.1 (Thermo Fisher Scientific).

### ﻿Phylogenetic analyses

*Hemiaustroboletus* species produce scarce fruit bodies; from 606 Boletales specimens collected, just eight (1.32%) belonged to this genus. Three materials corresponded to *H.vinaceus*, four to *H.vinaceobrunneus* and two were determined as *Hemiaustroboletus* sp. The three loci of the holotype of *H.vinaceus* (IBUG-AES334) and one more collection (ITCV-AV524, MEXU-30103) were sequenced; we only recovered ITS and RPB2 loci from a third specimen (IBUG-AES364) (Table 1). The three loci of the holotype of *H.vinaceobrunneus* (ITCV-AV868, MEXU-30051) and one additional material (ITCV-AV845, MEXU-30052) were sequenced; only the ITS and RPB2 loci were sequenced for a third collection (ITCV-AV1168, MEXU-30053). ITS locus was also sequenced for one *Hemiaustroboletus* sp. collection (ITCV-AK_3508) (Table 1).

**Table 1.** List of species, geographic origin and GenBank accession numbers of ITS, LSU and RPB2 sequences used in the phylogenetic analyses.

Taxa	Voucher	Country	ITS	LSU	RPB2	Reference
*Aureoboletusbetula*		USA		MK601736	MK766298	Kuo and Ortiz-Santana (2020)
*A.garciae*	MEXU:29006	Mexico		MH337251	MT228983	Haelewaters et al. (2020)
*Austroboletusamazonicus*	1839_ AMV	Colombia	KF937307	KF714508		Vasco-Palacios et al. (2014)
*A.amazonicus*	1914_ AMV	Colombia	KF937308	KF714509		Vasco-Palacios et al. (2014)
*A.austrovirens*	BRI:AQ0795791	Australia	KP242211	KP242225	KP242133	Fechner et al. (2017)
*A.austrovirens*	BRI:AQ0794622	Australia	KP242210			Fechner et al. (2017)
*A.austrovirens*	MEL:2382920a	Australia		KP242284	KP242113	Fechner et al. (2017)
*A.austrovirens*	BRI:AQ0794609	Australia		KP242226	KP242131	Fechner et al. (2017)
*A.austrovirens*	BRI:AQ0794171	Australia		KP242227	KP242133	Fechner et al. (2017)
*A.eburneus*	REH9487	Australia		JX889668		Vasco-Palacios et al. (2014)
*A.dictyotus*	HKAS59804	China		JX901138		Hosen et al. (2013)
*A.fusisporus*	HKAS75207	China	JX889719	JX889720		Hosen et al. (2013)
*A.fusisporus*	JXSB0351	China		MK765810		GenBank
*A.gracilis*	112-96	USA		DQ534624		Binder and Hibbett (2006)
*A.gracilis*	TM03_434	Canada		EU522815		Porter et al. (2008)
A.gracilisvar.gracilis	CFMR BOS-547	USA		MK601715	MK766277	Kuo and Ortiz-Santana (2020)
A.gracilisvar.flavipes	CFMR BOS-562	USA		MK601714		Kuo and Ortiz-Santana (2020)
*A.gracilis*	ACAD11344F	Canada	MH465078			Young et al. (2019)
*A.gracilis*	SFC20140823-02	South Korea	MN794901			GenBank
*A.gracilis*	NAMA 2017-106	USA	MH979242			GenBank
*A.gracilis*	310751	México	MH167935			GenBank
*A.gracilis*	CNV35	USA	MT345212			Victoroff (2020)
A.cf.gracilis	JLF6600	USA	MN174796			GenBank
*A.lacunosus*	REH9146	Australia		JX889669		Vasco-Palacios et al. (2014)
*A.lacunosus*	MEL2233764	Australia		KC552056		GenBank
*A.mucosus*	TH6300	Guyana		AY612798		Drehmel et al. (2008)
*A.mutabilis*	BRI:AQ0795793	Australia	KP242169	KP242263	KP242098	Fechner et al. (2017)
*A.mutabilis*	BRI:AQ0669270	Australia		KP242266	KP242097	Fechner et al. (2017)
*A.mutabilis*	BRI:AQ0796266	Australia		KP242262	KP242099	Fechner et al. (2017)
*A.niveus*	312	New Zealand		DQ534622		Binder and Hibbett (2006)
*A.niveus*	MEL2053830	Australia	KC552016	KC552058		Orihara et al. (2016)
*A.novae-zelandiae*	PDD:72542	New Zealand	HM060327			GenBank
*A.rarus*	BRI:AQ0794045	Australia	KP242197	KP242236	KP242086	Fechner et al. (2017)
*A.rostrupii*	TH8189	Guyana	JN168683			Smith et al. (2011)
*Austroboletus* sp.	BRI:AQ0794156	Australia		KP242235	KP242115	GenBank
*Austroboletus* sp.	BRI:AQ0794222	Australia		KP242234	KP242106	GenBank
*Austroboletus* sp.	BRI:AQ0794271	Australia		KP242259	KP242102	GenBank
*Austroboletus* sp.	HKAS 57756	China		KF112383	KF112764	Wu et al. (2014)
*Austroboletus* sp.	HKAS 59624	China		KF112485	KF112765	Wu et al. (2014)
*Austroboletus* sp.	HKAS 74743	China		KT990527	KT990367	Wu et al. (2014)
*Austroboletus* sp.	PERTH6658407	Australia		KP242277	KP242126	GenBank
*Austroboletus* sp.	BRI:AQ0794242	Australia			KP242087	GenBank
*Austroboletus* sp.	OR0891	Thailand			MH614753	Vadthanarat et al. (2019)
*Austroboletus* sp.	OTAFUNNZ2013434	New Zealand			KP191670	GenBank
*A.subflavidus*	JBSD130771	Dominican Republic		MT580902	MT590754	Gelardi et al. (2020)
*A.subflavidus*	JBSD130772	Dominican Republic		MT580903	MT590755	Gelardi et al. (2020)
*A.subflavidus*	CFMR BZ-3178	Belize		MK601716	MK766278	Kuo and Ortiz-Santana (2020)
*A.subvirens*	KPM-NC-0017836	Japan		JN378518		Orihara et al. (2012)
*A.viscidoviridis*	Perth 7588682	Australia		KP242282	KP242128	Fechner et al. (2017)
*Boletellusindistinctus*	HKAS77623	China		KT990531	KT990371	Wu et al. (2016)
*Boletellus* sp.	HKAS80554			KT990535	KT990374	Wu et al. (2016)
*Boletusharrisonii*	MICH: KUO-09071204	USA		MK601718	MK766280	Kuo and Ortiz-Santana (2020)
*Boletus* sp.	dd08055	China	FJ810161			GenBank
*Boletus* sp.	MHM165	Mexico	EU569243			Morris et al. (2008)
*Boletales* sp.	B0229	Canada	KY825985			GenBank
Fistulinellacampinaranaevar.scrobiculata	AMV1980	Colombia		KF714520		Vasco-Palacios et al. (2014)
*F.gloeocarpa*	JBSD130769	Dominican Republic		MT580906	MT590756	Gelardi et al. (2020)
*F.gloeocarpa*	CFMR:B4	Bahamas		MT580904		Gelardi et al. (2020)
*F.gloeocarpa*	CFMR:B10	Bahamas		MT580905		Gelardi et al. (2020)
*F.prunicolor*	REH9502	Australia		JX889648	MG212630	Halling et al. (2012)
*F.olivaceoalba*	HKAS 53432	Vietnam		MH745969		GenBank
*F.olivaceoalba*	LE312004	Vietnam		MH718396		GenBank
*F.ruschii*	CORT:TJB-8329	USA		MT580907		Gelardi et al. (2020)
*F.viscida*	238 25S	New Zealand		AF456826		Vasco-Palacios et al. (2014)
*F.cinereoalba*	TH8471	Guyana		GQ477439	KT339237	GenBank
***Hemiaustroboletusvinaceobrunneus***	**MEXU_30051 Holotype**	**Mexico**	**MN178797**	**MN200222**	**MT887617**	**This study**
***H.vinaceobrunneus***	**MEXU_30052 Isotype**	**Mexico**	**MN178798**	**MN200223**	**MT887618**	**This study**
***H.vinaceobrunneus***	**MEXU_30053 Isotype**	**Mexico**	**MN178799**		**MT887619**	**This study**
***H.vinaceus***	**AV524 Paratype**	**Mexico**	**MN178802**	**MN200225**	**MT887622**	**This study**
***H.vinaceus***	**AES334 Holotype**	**Mexico**	**MN178800**	**MN200224**	**MT887620**	**This study**
***H.vinaceus***	**AES364 Isotype**	**Mexico**	**MN178801**		**MT887621**	**This study**
***Hemiaustroboletus* sp.**	**AK_3508**	**Mexico**	**MN178803**			**This study**
*Hemileccinumsubglabripes*	MICH: KUO-08301402	USA		MK601739	MK766301	Kuo and Ortiz-Santana (2020)
*Hortiboletusrubellus*	MICH: KUO-06081002	USA		MK601741	MK766303	Kuo and Ortiz-Santana (2020)
*H.amygdalinus*	HKAS54166	China		KT990581	KT990416	Wu et al. (2016)
*Hourangiacheoi*	Tang572	China		KP136953	KP136985	Zhu et al. (2015)
*Imleriabadia*	MICH: KUO-09110404	USA		MK601743	MK766305	Kuo and Ortiz-Santana (2020)
*Mucilopiluscastaneiceps*	HKAS 75045	China		KF112382	KF112735	Wu et al. (2016)
*M.castaneiceps*	HKAS50338	China		KT990555	KT990391	Wu et al. (2016)
*M.castaneiceps*	HKAS71039	China		KT990547	KT990385	Wu et al. (2016)
*Parvixerocomuspseudoaokii*	HKAS 80480	China		KP658468	KP658470	Wu et al. (2016)
*Porphyrelluscastaneus*	HKAS52554	China		KT990697	KT990502	Wu et al. (2016)
*P.porphyrosporus*	MB97-023	Germany		DQ534643	GU187800	Binder and Hibbett (2006)
*P.orientifumosipes*	HKAS53372	China		KT990629	KT990461	Wu et al. (2016)
*Tengioboletus* sp.	HKAS 77869	China		KT990658	KT990483	Wu et al. (2016)
*Strobilomycesconfusus*	CFMR:DR-3024	Dominican Republic		MK601809	MK766365	Kuo and Ortiz-Santana (2020)
*Tylopilusfelleus*	CFMR: BOS-780	USA		MK601814	MK766370	Kuo and Ortiz-Santana (2020)
*T.sordidus*	MICH: KUO-06240801			MK601815	MK766371	Kuo and Ortiz-Santana (2020)
*Tylopilus* sp.	HKAS 50229	China		KF112423	KF112734	Wu et al. (2014)
Uncultured mycorrhizal	BOLETE1	USA	AY656925			Walker et al. (2005)
Uncultured mycorrhizal	clon N_1	South Korea	AB571507			Obase et al. (2012)
Uncultured *Boletus*	isolate: YM490	Japan	LC175482			Miyamoto et al. (2018)
Uncultured *Boletus*	Clon ZE2	China	GU391428			Ma et al. (2010)
*Veloporphyrellusalpinus*	KUN:HKAS68301	China		JX984537		Li et al. (2014)
*V.pseudovelatus*	KUN: HKAS59444	China		JX984542		Li et al. (2014)
*V.pseudovelatus*	KUN:HKAS52244	China		JX984531		Li et al. (2014)
*V.conicus*	CFMR:BZ1670	Belize		JX984543		Li et al. (2014)
*V.conicus*	CFMR:BZ1705	Belize		JX984544		Li et al. (2014)
*V.pantoleucus*	F:Gomez21232	Costa Rica		JX984548		Li et al. (2014)
*V.velatus*	KUN: HKAS63668	China		JX984546		Li et al. (2014)
V.aff.velatus	HKAS 57490	China		KF112380	KF112733	Wu et al. (2014)
*V.vulpinus*	LE315544	Vietnam	MN511177	MN511170		GenBank
*V.vulpinus*	LE315549	Vietnam	MN511180			GenBank
*V.vulpinus*	LE315546	Vietnam	MN511179			GenBank
*V.vulpinus*		Vietnam	MN511178			GenBank
*Xerocomelluschrysenteron*	HKAS:56494	China		KF112357	KF112685	Wu et al. (2014)

We conducted two sets of phylogenetic analyses, the first one to reconstruct the phylogenetic relationships of *Hemiaustroboletus* gen. nov. and the second one to complement its taxonomic concept with biogeographic and ecological information. The first analysis used the LSU and RPB2 markers in a concatenated matrix, while the second used ITS in order to leverage GenBank data.

Individual LSU and RPB2 alignments were concatenated into a single matrix (83 taxa, 1335 characters) with GENEIOUS PRIME V.2019.0.4 (Biomatters Ltd). Alignments and concatenation were performed with the MAFFT algorithm (Katoh et al. 2002) using GENEIOUS PRIME V.2019.0.4. Sequences representing the subfamilies Austroboletoideae, Boletoideae and Xerocomoideae came from: 83 LSU sequences, 56 rpb2 sequences, 30 ITS sequences from published works and unpublished sequences available in GenBank (Table 1).

The best-fit evolutionary model was estimated with JMODELTEST 2 (Darriba et al. 2012) using CIPRES SCIENCE GATEWAY V. 3.3 (Miller et al. 2010) for each marker separately. For all three markers, the best model was GTR+G+I. We used the LSU-RPB2 dataset to make evolutionary inferences within Austroboletoideae and the ITS dataset to make biogeographic/ecological inferences for *Hemiaustroboletus*.

The phylogenetic hypotheses (LSU-RPB2) were constructed with Bayesian Inference (BI) and Maximum Likelihood (ML) on a partitioned alignment with same evolutionary model for both markers. Bayesian posterior probability phylogeny was performed using MrBayes algorithm (Ronquist et al. 2012) using two separate Monte Carlo four chains starting from random trees for 10 million generations each (final standard deviation ± 0.224), trees were sampled every 100 generations. The first 25% of samples were discarded as burn-in. ML analyses were performed using the RAxML algorithm (Stamatakis 2014) with 1000 bootstrap replicates. For both analyses, members of subfamilies Boletoideae and Xerocomoideae were used as outgroup. The second analysis (ITS) was performed with the same parameters including *Veloporphyrellus* and *Austroboletus* without outgroup. The resulting phylogenetic trees were edited with FIGTREE V.1.4.3 (Rambaut 2009).

Average intrageneric and intergeneric nucleotide similarities between the genera within Austroboletoidеae were obtained separately for RPB2, LSU and ITS alignments as follows. For each alignment a nucleotide similarity matrix was computed in GENEIOUS 10.2.6 (Biomatters Ltd). Sequences belonging to genera outside Austroboletoidеae were removed and then the mean nucleotide similarity was calculated amongst all pairwise comparisons between sequences of each pair of genera.

## ﻿Results

**Figure 1.**
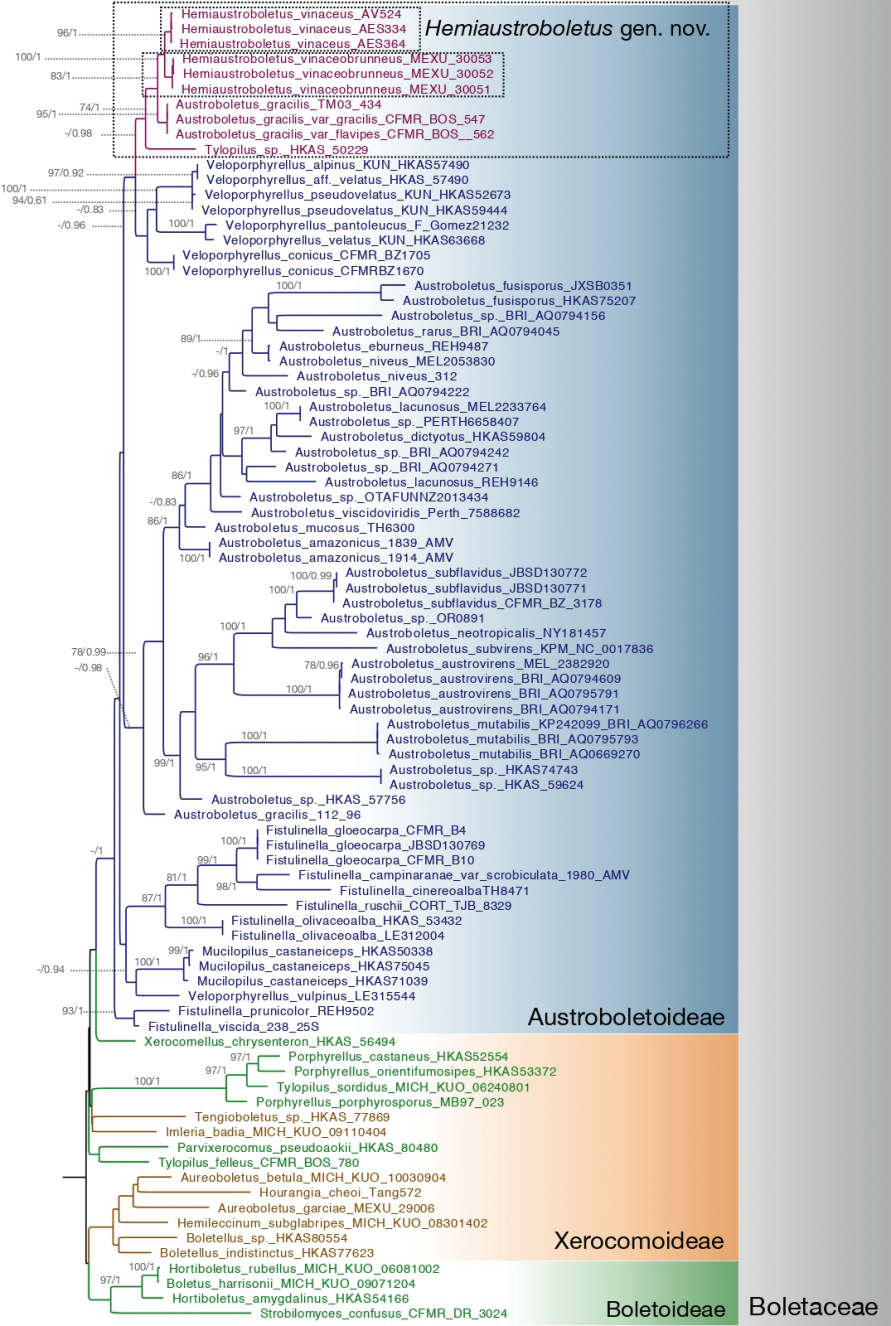
Phylogenetic placement of *Hemiaustroboletus* gen. nov. in the Austroboletoideae subfamily (Boletaceae) using LSU and RPB2 markers in a concatenated and partitioned matrix. The tree shows the topology of Bayesian analysis, with both MLB (≥ 70%) and BPP (≥ 0.7) clade support given. New genera and new species are indicated in the rectangles; taxa and/or branches in purple correspond to *Hemiaustroboletus* gen. nov.; remaining Austroboletoideae (blue); Boletoideae (green); Xerocomoideae (mustard). Background colours correspond to subfamilies; grey bars correspond to families.

**Figure 2.**
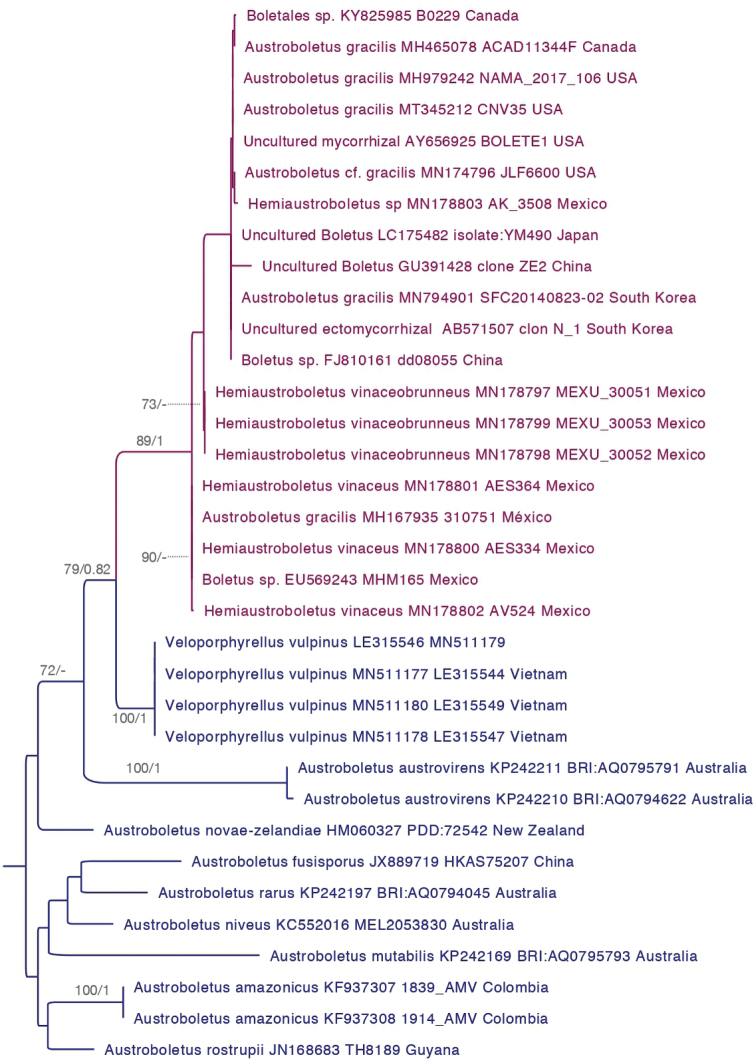
Phylogenetic tree of *Hemiaustroboletus* displaying geographic distribution using voucher and environmental ITS nrDNA sequences. The tree shows the topology of Bayesian analysis, with both MLB (≥ 70%) and BPP (≥ 0.7) clade support given. Taxa and branches in purple correspond to *Hemiaustroboletus* gen. nov. and those in blue to *Veloporphyrellus* and *Austroboletus*.

Phylogenetic analyses of LSU-RPB2 concatenated alignment showed that *Hemiaustroboletus* is a supported monophyletic group, belonging to the Austroboletoideae (BPP = 0.98, MLB = 47%). Additionally, *H.vinaceobrunneus* (BPP = 1, MLB = 100%) and *H.vinaceus* (BPP = 1, MLB = 96%) were supported monophyletic species (Fig. 1). The ITS analyses showed that *Hemiaustroboletus* forms ectomycorrhizae with Fagaceae, particularly *Quercus* and also with *Pinus* in temperate, subtropical and tropical forests. It distributes in North America (Mexico, USA and Canada) and Asia (China, Japan and Korea) (Fig. 2). These analyses also showed that *Austroboletusgracilis* s.l. is a widely-used name mainly applied to designate *Hemiaustroboletus* species.

### ﻿Taxonomy

#### ﻿ Hemiaustroboletus

Taxon classificationFungiBoletalesBoletales

Ayala-Vásquez, García-Jiménez & Garibay-Orijel gen. nov.

25454D0D-0AD2-5C47-BA68-0C6023708B39

 838460

##### Diagnosis.

*Hemiaustroboletus* is characterised by small and medium basidiomata with slightly ornamented pileus surface, stipe fibrillose to striated without veil, slightly verrucose or cracked to pitted basidiospores and pileipellis formed by an ixotrichoderm or trichoderm.

##### Etymology.

From the Latin *hemi* “almost or half”, *Austroboletus* the generic epithet refers to the morphological affinities with this genus.

##### Generic type.

*Hemiaustroboletusvinaceobrunneus* Ayala-Vásquez, García-Jiménez & Garibay-Orijel sp. nov.

##### Generic Description.

Epigeous, stipitate-pileate basidiomata. **Pileus** reddish-brown, violet-brown, dark violet, reddish-brown, orange-brown, yellow-brown, cinnamon, dry surface, finely velvety, velutinous, rivulose, granular-tomentose, subtomentose, minutely areolate. **Hymenophore** tubular, circular to angular pores, whitish, pink-purple, lilac, magenta-grey, brown-violet to pinkish-brown, with or without change brown when cut. **Context** whitish to pale red. **Stipe** subclavate, tomentose, pruinose, granular furfuraceous, striate surface, longitudinally fibrous, very finely reticulated in tapering towards apex. Whitish basal mycelium. **Basidiospores** ornamented, slightly verrucose, cracked to pits, fusoid, oval-elliptical, cylindrical to subfusoid, oblong, ovoid-oblong. **Cystidia** clavate, sphaeropedunculate, subfusoid. **Pileipellis** an ixotrichoderm or trichoderm; terminal cells cylindrical, fusoid, ventricose-rostrate with or without encrustations in the wall. **Caulocystidia** fusoid, cylindrical to subclavate and tetrasporic caulobasidia.

##### Distribution.

Canada, China, Japan, Mexico, South Korea and United States.

##### Ecology.

Temperate and subtropical forests, with conifers and broadleaf trees (*Abies* spp., *Quercus* spp., *Pinus* spp.) from 2000 to 3000 m alt.

#### ﻿ Hemiaustroboletus vinaceobrunneus

Taxon classificationFungiBoletalesBoletales

Ayala-Vásquez, García-Jiménez & Garibay-Orijel sp. nov.

599CA15C-FBF9-5630-97C3-F44398669F7B

 838461

Figs 3 , 4 , 5B, D

##### Diagnosis.

Pileus vinaceous to brown, pores whitish to pinkish at maturity, vinaceous context; longitudinally fribrillose stipe; basidiospores (10) 11–17 (–21) × 4–5 (–7) µm, slightly verrucose to cracked, fusoid to cylindrical; pleurocystidia ventricose-rostrate to fusoid, cheilocystidia sphaeropedunculate.

**Figure 3.**
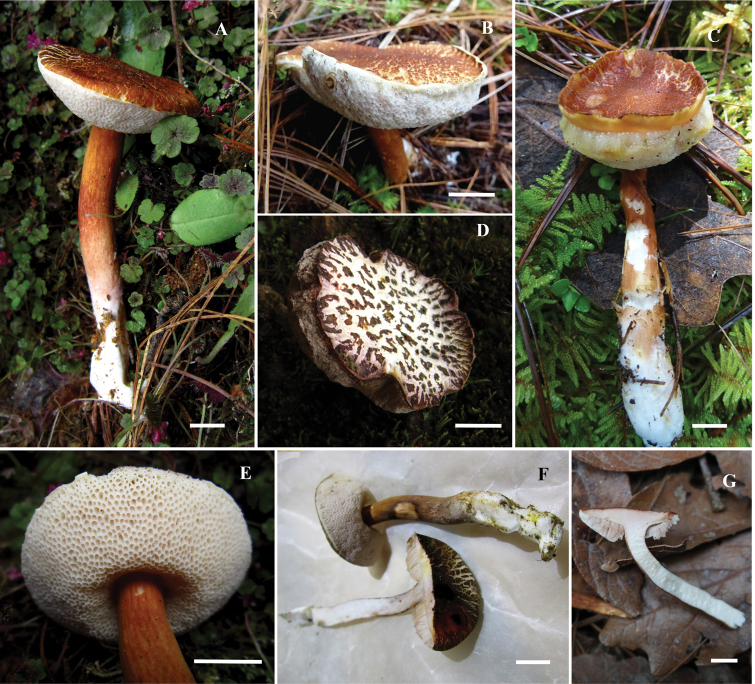
*Hemiaustroboletusvinaceobrunneus***A, C** basidiomata (MEXU-30052 Holotype) **B, D** pileus (MEXU-30053, MEXU-30051, Isotype) **E** hymenophore (MEXU-30052 Holotype) **F, G** context (MEXU-30052 Holotype). Scale bar: 10 mm (**A–G**).

##### Holotype.

Mexico. Oaxaca State, Santa Catarina Ixtepeji Municipality, La Cumbre Town, Peña Prieta site, 17°11'11.34"N, 96°38'00"W (DMS), 2800 m alt., 19 July 2017, Ayala-Vásquez (MEXU-30051; isotype ITCV-AV868).

**Figure 4.**
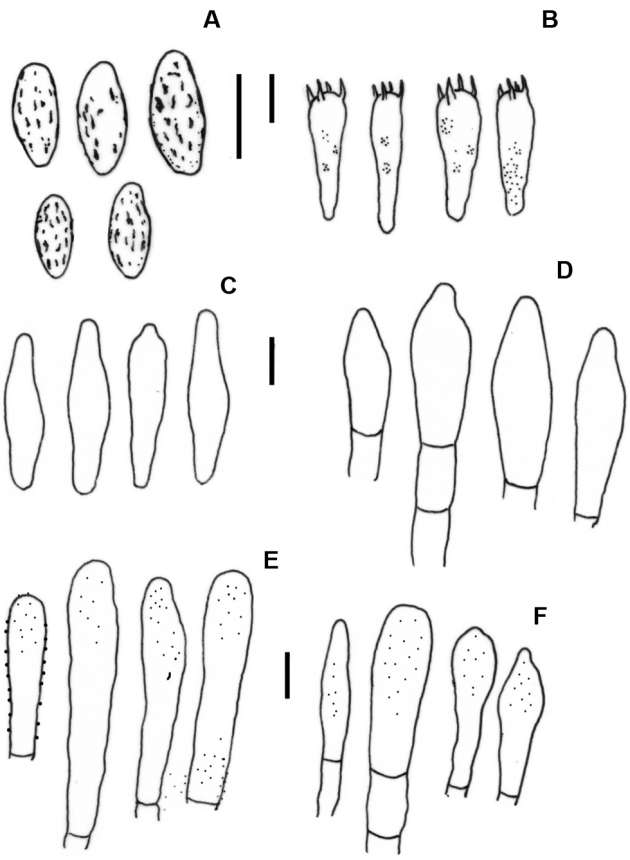
*Hemiaustroboletusvinaceobrunneus* (AV845-ITCV, MEXU-30052 Holotype) **A** basidiospores **B** basidia **C** pleurocystidia **D** cheilocystidia **E** pileipellis **F** caulocystidia. Scale bars: 10 µm (**A–F**).

##### Etymology.

The name refers to the colour of the pileus, from the Latin “*vinosus*” vinaceous when young and “*brunneus*” brown when mature.

**Figure 5.**
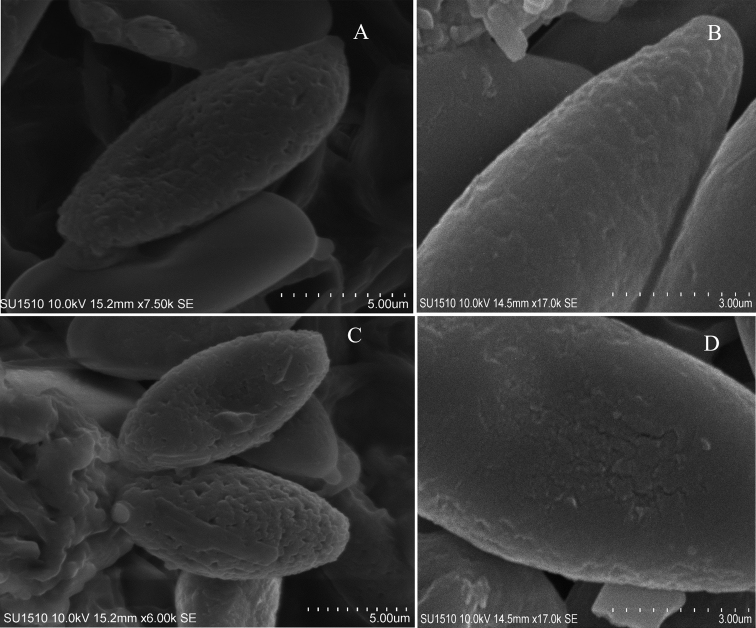
Basidiospore ornamentation of *Hemiaustroboletus* revealed by SEM**A, C***Hemiaustroboletusvinaceus* (AV868-ITCV, MEXU-30051, Holotype) **B, D***Hemiaustroboletusvinaceobrunneus* (AV1168-ITCV, MEXU-30053 Isotype).

##### Description.

Basidiomata stipitate-pileate. **Pileus** 36–40 mm diameter, convex when young becoming plano-convex, reddish-vinaceous (13B6) when young, orange brown (7C8), reddish-brown (8D8-8E8) to dark brown (7F8) with some ruby tones (12E8) at maturity, dry surface, subtomentose, rivulose to areolate, whitish context, decurved margin. **Hymenophore** slightly depressed around the stipe to subadnate, pores 1–1.2 mm diameter, circular to subangular, whitish when young, pink to red-whitish (11A3-11A2) at maturity, tubes 6 mm length, of pores concolorous, unchanging when cut or touched, tubes detachable from the context. **Context** 4–8 mm thick, whitish, with some shades of pale red, vinaceous at the edge of the pileus and at the apex of the stipe at maturity. **Stipe** 45–65 × 8–10 mm, subclavate, reddish-vinaceous (13B6), orange-brown (7C8) to brown (7D8 -7E8) at the apex and part of the base, orange in the middle area (6B8) to orange-brown (6C8), rest of the base whitish; surface furfuraceous, longitudinally fibrillose. Whitish mycelium. **Chemical reactions** pileus negative in KOH, the context and the hymenophore slightly become pale violet (16A2) and the stipe becomes pale brown (6D4). When ammonium hydroxide (NH_4_OH) is applied, the pileus becomes brown-violet (11F8-11F7), the hymenophore and context pale orange (5A2) and the stipe pale violet (16A2).

**Basidiospores** 10–15 (–20) × 4–5 (–7) µm, X = 14.04 × 4.96 µm, std = 3.46 × 0.99 µm, (n = 30, Q = (2.2) 2.4–2.5 (2.8), (holotype); (10–) 11–15 (–21) × 4.5–7 (–8) µm, X = 13.78 × 6.07 µm, std = 3.74 × 1.3 µm, Q = (2.2) 2.4–2.6 (2.8) (paratype MEXU-30052); (10–) 11–15 (–17) × (4–) 4.5–5.5 (–6) µm, X = 13.15 × 4 µm, std = 2.62 × 0.64 µm, Q = (2.2) 2.6–2.9 (3) µm, (paratype ITCV-AV1121), cylindrical to subfusoid, slightly verrucose to cracked, brown-orange in KOH, inamyloid in Melzer’s reagent. **Basidia** 30–33 (–49) × 9–11 (–12) µm, clavate, hyaline in KOH, pale yellow in Melzer’s reagent, with granular content, tetrasporic. **Pleurocystidia** 31–45 × 8–11 µm, ventricose to fusoid, some mammillate, hyaline in KOH, yellowish in Melzer’s reagent, thick walled (1–1.5 µm). **Cheilocystidia** 42–70 (–86) × 9–15 (–17) µm, clavate with septa (1–2 µm thick), sphaeropedunculate, some mammillate, hyaline in KOH, yellowish in Melzer’s reagent, thick-walled (1–1.5 µm). **Hymenophoral trama** boletoid; hyphae cylindrical 3–15 µm diameter, with gelatinous wall some with smooth walls, hyaline to yellowish in KOH and Melzer’s reagent. **Pileipellis** a trichoderm with terminal cells (22–) 35–75 (–105) × 8–14 (–21) µm, cylindrical to subclavate, hyaline in KOH, yellowish in Melzer’s reagent, embedded in a gelatinous substance and with visible contents in Melzer’s reagent, thick-walled (1–1.5 µm). **Caulocystidia** 20–64 (–140) × 6–14 (–16) µm, fusoid, cylindrical to sphaeropedunculate with one to two septa, hyaline to yellowish KOH with visible contents visible in Melzer’s reagent. **Caulobasidia** 25–30 × 7–8 µm tetrasporic, concolorous with the caulocystidia. **Clamp connections** absent.

##### Habit and habitat.

Solitary, in *Abiesguatemalensis*, *Pinuspseudostrobus* and *Quercuslaurina* mixed forest, putatively associated with *Quercuslaurina*, from 2800 to 3000 m alt.

##### Known distribution.

Currently only known from Oaxaca State, southeast Mexico.

##### Additional materials examined.

Mexico, Oaxaca State, Santa Catarina Ixtepeji Municipality, La Cumbre Town, East of cottage site, 17°11'30"N, 96°38'18"W (DMS), 2903 m alt., 18 July 2017, Ayala-Vásquez (MEXU-30052; ITCV-AV845); Cabeza de Vaca site, 17°11'10"N, 96°38'28"W (DMS), 3038 m alt., 18 July 2017, Ayala-Vásquez (ITCV-AV1121), Cabeza de Vaca site, 15 August 2018, Ayala-Vásquez (MEXU-30053; ITCV-AV1168).

##### Remarks.

*Hemiaustroboletusvinaceobrunneus* differs from *H.vinaceus* by its context with vinaceous tones especially at maturity and a whitish-pink to pale red hymenophore; the stipe is orange-brown; basidiospores are 10–15 (–20) × 4–5 (–7) µm, finely verrucose to cracked, lodged to sphaeropedunculate cheilocystidia, caulocystidia fusoid, cylindrical to sphaeropedunculate with a septum. In contrast, *H.vinaceus* has a whitish context with slight yellowish-brown tones near the epicutis, has shorter basidiospores (9–) 10–14.4 (–16) × 4–5(–8) µm, cylindrical to clavate queilocystidia and caulocystidia fusoid or clavate. In the field, the former can be mistaken for *Gyroporuspurpurinus* because of the colours and size of the basidiomata, but *G.purpurinus* has a hollow stipe (Davoodian and Halling 2013), while *H.vinaceobrunneus* has a compact context.

#### ﻿ Hemiaustroboletus vinaceus

Taxon classificationFungiBoletalesBoletales

Ayala-Vásquez, García-Jiménez & Saldivar sp. nov.

2FB86FAE-4429-5AE6-8F7A-497983D4DC03

 838462

Figs 5A, C , 6 , 7

##### Diagnosis.

Pileus dark violet to dark brown, whitish context; hymenophore pink-purple to violet-brown; stipe surface tomentose to longitudinally fribrillose; basidiospores 9–13 × 4–5 µm, surface with cylindrical pits; pleurocystidia and cheilocystidia fusiform-ventricose to lanceolate.

**Figure 6.**
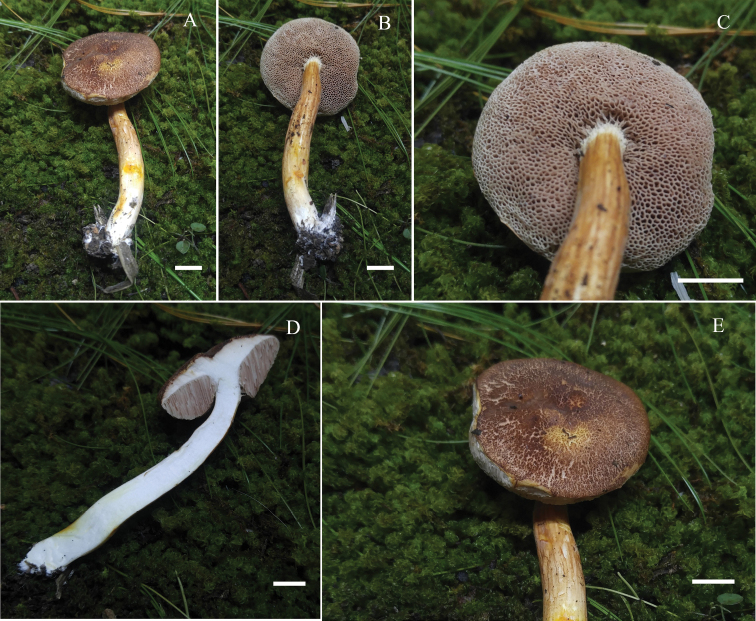
*Hemiaustroboletusvinaceus* (AES334-IBUG, Holotype) **A, B** basidiomata **C** hymenophore **D** context **E** pileus surface. Scale bar: 10 mm (**A–E**).

##### Holotype.

Mexico, Jalisco State, Tequila Municipality, Tequila Volcano site, between 11 and 12 km on the road uphill to the antenna station, 20°48'35"N, 103°51'46"W (DMS), 2144 m alt., 18 August 2019, Á.E. Saldivar (IBUG-AES334).

**Figure 7.**
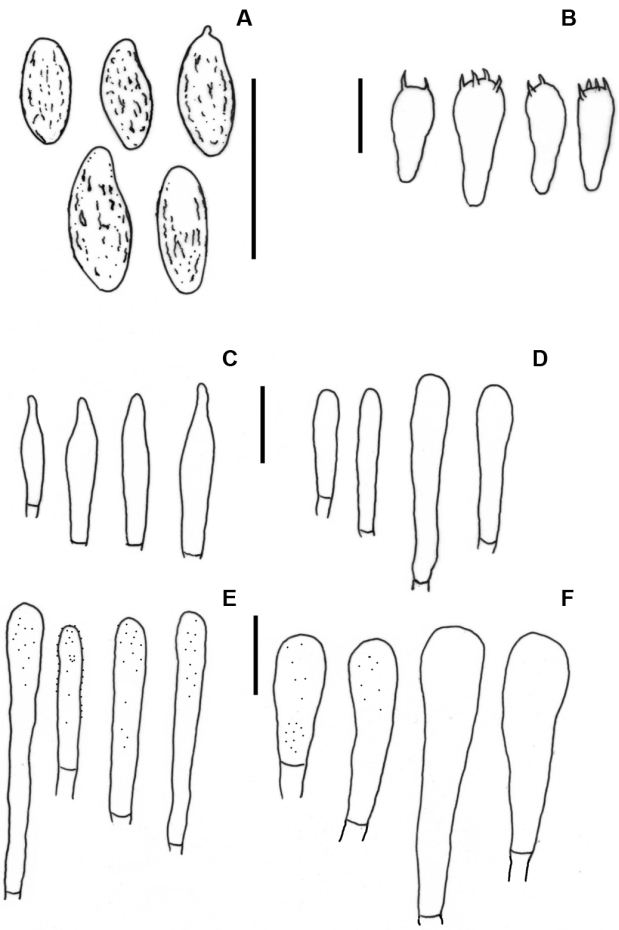
*Hemiaustroboletusvinaceus* (AES334-IBUG, Holotype) **A** basidiospores **B** basidia **C** pleurocystidia **D** cheilocystidia **E** pileipellis **F** caulocystidia. Scale bars: 10 µm (**A–F**).

##### Etymology.

The name refers to the colour of the pileus from the Latin “*vinosus*” vinaceous.

##### Description.

**Pileus** 35–70 mm in diameter, convex when young, becoming plano-convex with age, dark violet (16F6-16F4), violet-brown (11F5-11F8), orange-brown (5E7), with lighter shades of dark brown (6F5-6F8) lighter towards margin, whole edge, straight, dry surface, finely scamose, slightly areolate at the centre. **Hymenophore** adnate, slightly depressed, pores 0.5–2 mm in diameter, subangular to angular, pink-purple (14A4), lilac (14B4–14C4), magenta-grey (14C4–14D4), ruby-grey (12C4–12D4), colour unchanging when injured, tubes 7–10 mm, concolorous with the pores. **Context** 7–12 mm thick, solid, whitish, with slight yellowish-brown tones near the epicutis. **Stipe** 62–77 × 8–9 mm, central, cylindrical, with wider base, surface with longitudinal striations, whitish at the apex, yellowish-brown (5D5-5E5), orange-brown (5C5) shades in the middle, base with yellowish (5B6) to whitish shades; whitish context, unchanged when cut. Whitish basal mycelium. **Odour** pleasant. **Taste** slightly acidic. **Chemical reactions**: KOH reddish-brown in pileus, brown in hymenophore, slightly pinkish in context, yellowish-brown in stipe. NH_4_OH orange with violet tones on pileus, yellow in hymenophore, pale yellow in context, red-orange in stipe.

**Basidiospores** 9–13 (–14.5) × 4–5 (–8) µm, X = 12.14 × 5.2 µm, std = 2.08 × 1.36 µm, (n = 35), Q = (1.8) 2.1–2.2 (2.5) (holotype); (10–) 12–14 × 4–5 (–7) µm, X = 11.94 × 5.14 µm, std = 1.60 × 1.13 µm, (n = 35), Q = (2.2) 2.3–2.4 (2.5), (paratype MEXU-30103); (10–) 14–15 (–16) × (4–) 5–6 (–7) µm, X = 14.29 × 5.8 µm, std = 1.69 × 0.76 µm, (n = 40), Q = (2.2) 2.3–2.5 (2.6), (paratype colpos-CP5); subfusiform to cylindrical, slightly rough or dotted, apex rounded to subacute, with suprahilar depression, yellowish. **Basidia** 27–34 × 7–15.2 µm, claviform, bisporic, tetrasporic, with sterigma 2–4 × 0.5–1 µm, thin-walled, hyaline in KOH, yellow in Melzer’s reagent. **Pleurocystidia** 28–50 × 6.4–11 µm, fusoid-ventricose, slightly lanceolate, with content hyaline in KOH, yellow in Melzer’s reagent, with walls 0.5 μm thick. **Cheilocystidia** 25–61 × 6.4–11 µm, subclavate, hyaline in KOH, yellow in Melzer’s reagent, thin-walled. **Hymenophoral trama** divergent, with central and lateral hyphae tubular, 2–6 µm wide, hyaline in KOH, yellow in Melzer’s reagent, thin-walled; septa without clamp connections. **Pileipellis** a trichoderm with terminal cells 32–92 × 5–11 µm, cylindrical to subclaviform, hyaline in KOH, yellow in Melzer’s reagent, thin-walled. **Caulocystidia** 29–95 × 14–17 (–19) µm, subclaviform to claviform, thin-walled, with yellow visible contents in Melzer’s reagent, hyaline in KOH.

##### Habit and habitat.

*Pinus*-*Quercus* forests and *Quercus* forests, associated with *Q.liebmanii* and other *Quercus* spp.

##### Known distribution.

Currently only known from Neovolcanic Axis and Sierra Madre del Sur, Mexico.

##### Additional material examined.

Mexico, Jalisco State, Tequila Municipality, Tequila Volcano site, km 11–12 on the road uphill to the antenna station, 20°48'14"N, 103°51'37"W (DMS), 2144 m alt., 18 September 2019, A.E. Saldivar (IBUG-AE364); Oaxaca State, San Antonio de la Cal Municipality, Las Peñas site, 17°01'11"N, 96°40'33"W (DMS), 2160 m alt., 4 October 2014, Ayala-Vásquez (MEXU-30103; ITCV-AV524, duplicated ENCB); Michoacan State, Road Morelia, Ciudad Hidalgo Town, km 40, 21 July 1983, García-Jiménez (ITCV-3662), Mil Cumbres Town, 9 August 1969, R. Singer M8993 (F). Estado de México State, Ocuilan, San Juan Atzingo Town, mixed forest, 15 July 2021, mycoredes (Colpos- CP5).

##### Remarks.

*Hemiaustroboletusvinaceus* differs from *H.vinaceobrunneus* due to its dark violet pileus, lilac to violet hymenophore, yellow stipe in the basal area and whitish apex. It has short, perforated basidiospores 9–13 (–14.4) × 4–5 (–8) µm, caulocystidia clavate to fusoid and pileipellis formed by a trichoderm with terminal cell cylindrical or subclavate, thin-walled. In contrast, *H.vinaceobrunneus* has a pileipellis formed by a trichoderm with encrustations. *Hemiaustroboletusvinaceus* is easily confused with *Austroboletusgracilissensu*Wolfe (1979), because of its macroscopic characteristics and basidiospore ornamentation, but *A.gracilis* differs by pileus red-brown, brown-orange, having a total or partial reticulum on the stipe surface; longer basidiospores 10–19.5 × 4.5–9 µm, rugulose- punctate, elliptical to ovoid-elliptical. Austroboletusvar.gracilis (Peck) Wolfe differs from *H.vinaceus* by pileus surface dry, finely velvety, when young, sometime rimose, reddish-brown, cinnamon or yellow-brown; stipe surface anastomosing lines, narrow reticulation overall or at least on the upper half; basidiospores 10–17 × 5–8 µm, narrowly ovoid to subelliptical. Austroboletusgracilisvar.laevipes is distinguished by the smooth stipe, pileus yellow-ochraceous to yellow-brown, stipe subclavate, striate, finely pruinose, neither ribs nor reticulated surface, pale yellow or yellow-brown, basidiospores 11.2–14 × 5–8µm, oval-elliptical in face view, inequilateral in profile (Bessette et al. 2000). Austroboletusgracilisvar.pulcherripes Both & Bessette differs from *H.vinaceus* by a white hymenium when young, becoming pinkish to pale cocoa at maturity; stipe clavate, surface dry, coarsely reticulated on the upper two- thirds, reticulated, finely tomentose; basidiospores 13–19 × 5–8 µm, smooth to rugose-punctate, ovoid-elliptical, narrowly ovoid, inequilateral profile.

## ﻿Discussion

According the phylogenetic analysis, our collections are nested within the Austroboletoideae close to *Veloporphyrellus*. Recognising the *Hemiaustroboletus* genus contributes to solving the systematics within Austroboletoideae since previous works have shown that *Austroboletus* and *Veloporphyrellus*, as currently morphologically circumscribed, are polyphyletic (Wu et al. 2016; Gelardi et al. 2020; Kuo and Ortiz-Santana 2020). For example, Wu et al. (2016) found two clades of *Austroboletus*, *Austroboletus*. s.s. and a second clade where *Austroboletusgracilis* s.l. (strain, 112/96) is nested with *Veloporphyrellusgracilioides*, this species being separated from the *Veloporphyrellus* s.s. clade. Gelardi et al. (2020) also recovered *Austroboletus* as polyphyletic with *Austroboletus* s.s. containing most of the species and other samples divided into four more clades. Particularly, in their analyses, most *A.gracilis* samples nested close to *Veloporphyrellus*; this is the clade we are erecting now as *Hemiaustroboletus*.

Our analyses show that *Hemiaustroboletus* is related to *Veloporphyrellus* (Fig. 1). This is supported by previous analyses (Gelardi et al. 2020; Kuo and Ortiz-Santana, 2020); indeed, they differ in several morphological characteristics. *Veloporphyrellus* has a veil which often embraces the apex of the stipe in younger basidiomata; hymenophoral surface white when young becoming pinkish to pink when mature; basidiospores smooth subfusiform to oblong. In contrast, *Hemiaustroboletus* has furfuraceous, tomentose to minutely areolate pileus surface; whitish, pink-purple, lilac, magenta-grey to brown-violet hymenophoral surface; and slightly verrucose, cracked to pitted ornamented basidiospores (Table 2). Even while the phylogenetic relations between both genera are not statistically supported, nucleotide similarity demonstrated that they are the closest genera within Austroboletoidеae. The overall nucleotide similarity between genera in Austroboletoidеae in RPB2 is 89.23%, in LSU it is 88.19%, and in ITS it is 72.55%. Between *Hemiaustroboletus* and *Veloporphyrellus*, the average nucleotide similarity is 93.45% in RPB2, 94.01% in LSU and 74.75 in ITS (Table 3). These amounts of variation in the three markers also support the conclusion of recognising both genera.

**Table 2.** Comparative table of Austroboletoidеae genera, based on Wolfe (1979) and Wu et al. (2016).

Genera	Basidiomata	Basidiospores	Cystidia	Pileipellis
***Austroboletus***	Pileus margin which embraces the stipe when young. Stipe surface distinctly reticulate, alveolate-lacunose	Ornamented, elongate to amygdaliform, with warts, reticulate ridges or shallow to irregularly furrowed pits	Cylindrical, clavate, fusoid	Trichoderm with filamentous interwoven hyphae, sometimes strongly gelatinous
***Fistulinella***	Stipitate-pileate to occasionally sequestrate, with or without veil, usually viscid to strongly glutinous pileus	Smooth, elongate fusoid, inamyloid to dextrinoid	Fusiform to ventricose fusiform or lageniform	Trichoderm, ixotrichoderm or ixocutis
***Hemiaustroboletus***	Pileus surface furfuraceous, tomentose, minutely areolate, stipe surface longitudinally fibrillose to striate	Slightly verrucose, cracked to pitted	Clavate, Ropedunculate, subfusoid	Ixotrichoderm or trichoderm, terminal cells cylindrical, fusoid, ventricose-rostrate
***Mucilopilus***	Viscid pileus, stipe without colour change, white to pinkish or pink hymenophore	Smooth, subfusiform to oblong	Fusoid, ventricose to subfusiform	Ixotrichoderm, composed of strongly gelatinous filamentous hyphae
***Veloporphyrellus***	Pileus margin with distinct membranous veil or appendiculate, stipe nearly glabrous or fibrillose	Smooth, subfusiform to oblong	Subfusiform to ventricose	Trichoderm composed of filamentous interwoven hyphae

*Hemiaustroboletus* gen. nov. accomplishes the guidelines for the establishment of new genera proposed by Vellinga et al. (2015). It is a monophyletic group supported by morphological data and phylogenetic analyses (BPP = 0.98) (Fig. 1). When *Hemiaustroboletus* is recognised, the related clade *Austroboletus* s.s. (the clade including *A.dictyotus*, the genus type) becomes monophyletic. Additionally, the DNA sequence sampling is broad in taxonomic and geographic terms and uses ribosomal markers and protein coding genes. Indeed, holotypes for both species described are represented with the three markers included in the phylogenetic analyses.

**Table 3.** Average nucleotide similarity amongst genera of Austroboletoidеae.

Genus 1	Genus 2	Average nucleotide similarity (ITS) %	Average nucleotide similarity (LSU) %	Average nucleotide similarity (RPB2) %
***Hemiaustroboletus***	***Hemiaustroboletus***	**95.49**	**98.93**	**97.96**
***Hemiaustroboletus***	***Mucilopilus***		**92.51**	**91.25**
***Hemiaustroboletus***	***Austroboletus***	**71.27**	**85.94**	**87.75**
***Hemiaustroboletus***	***Fistulinella***		**88.58**	**89.76**
***Hemiaustroboletus***	***Veloporphyrellus***	**74.75**	**94.01**	**93.45**
*Veloporphyrellus*	*Veloporphyrellus*		95.49	100
*Veloporphyrellus*	*Austroboletus*		85.64	86.66
*Veloporphyrellus*	*Mucilopilus*		91.45	89.73
*Veloporphyrellus*	*Fistulinella*		88.06	89.5
*Fistulinella*	*Fistulinella*		90.48	89.5
*Fistulinella*	*Mucilopilus*		87.61	89.5
*Fistulinella*	*Austroboletus*		83.03	86.87
*Austroboletus*	*Austroboletus*		86	92.06
*Austroboletus*	*Mucilopilus*		85.05	87.88
*Mucilopilus*	*Mucilopilus*		98.5	99.4

*Hemiaustroboletus* is proposed as a new genus with two species *H.vinaceobrunneus* and *H.vinaceus*, including several of the revised material being previously identified as *A.gracilis* by Singer et al. (1991), Ayala-Vásquez et al. (2018) and Saldivar et al. (2021). The genus has at least one more known clade (Fig. 1) containing samples originally identified as *A.gracilis* (TM03-434) from Canada, A.gracilisvar.gracilis (CFMR BOS-547) and A.gracilisvar.flavipes (CFMR BOS-562) from USA. As found in our analyses and previous works (Wu et al. 2016; Gelardi et al. 2020; Kuo and Ortiz-Santana 2020), *A.gracilis* is a name widely applied to several clades. In our analysis, the sample *A.gracilis* 112/96 belongs to *Austroboletus* (maybe because it lacks RPB2 locus), while the rest of the sequences with this epithet belong to *Hemiaustroboletus*. As this species is polyphyletic, establishing the true identity of *A.gracilis* s.s. requires the sequencing of its type specimen, a task beyond the objectives of this study.

*Hemiaustroboletus* differs morphologically from Austroboletussect.Austroboletus sensu Wu et al. (2016) (*Austroboletus* s.s. in this study) because the species of the latter have clearly reticulated to costate stipe, elongate, fusoid or amygdaliform basidiospores with warts, reticulate ridges, irregularly furrowed pits or shallow ornamentation and a subrepent to trichoderm pileipellis, composed of filamentous interwoven hyphae, sometimes strongly gelatinous. In contrast, *Hemiaustroboletus* is characterised by a subclavate, tomentose, pruinose, granular furfuraceous, striate surface, longitudinally fibrous, very finely reticulated stipe, oval-elliptical, cylindrical to subfusoid, oblong, ovoid-oblong basidiospores with slightly verrucose, cracked to pitted surface, its pilleipellis is an ixotrichoderm or trichoderm with terminal cells cylindrical, fusoid or ventricose-rostrate with or without incrustations in the wall.

Finally, *A.gracilis*, described by Ortiz-Santana et al. (2007) from Central America, is probably *Hemiaustroboletusvinaceus* or a close species, because they match the description presented here. Further analysis of these collections and others, labelled as *A.gracilis* in subtropical regions of Central America and eastern Asia, are needed to fully understand the diversity and distribution of *Hemiaustroboletus*.

## Supplementary Material

XML Treatment for Hemiaustroboletus

XML Treatment for Hemiaustroboletus vinaceobrunneus

XML Treatment for Hemiaustroboletus vinaceus
